# Measurements of the swimming speeds of motile microorganisms using object tracking and their correlation with water pollution and rheology levels

**DOI:** 10.1038/s41598-021-91134-1

**Published:** 2021-06-03

**Authors:** Ashaa Preyadharishini Shunmugam, Gowtham Subramanian, Javier G. Fernandez

**Affiliations:** 1 Pillar of Engineering Product Development (EPD), Singapore University of Technology and Design (SUTD), Singapore, 487372 Singapore; 2 Skin Research Institute of Singapore, Agency for Science Technology and Research, Singapore, 138648 Singapore

**Keywords:** Biochemical assays, Environmental monitoring

## Abstract

Self-propelled microscopic organisms are ubiquitous in water. Such organisms’ motility depends on hydrodynamic and physical factors related to the rheology of the surrounding media and biological factors depending on the organisms’ state and well-being. Here we demonstrate that the swimming speed of *Paramecium aurelia*, a unicellular protozoan, globally found in fresh, brackish, and salt waters, can be used as a measurable frugal indicator of the presence of pollutants in water. This study establishes a significant and consistent relationship between *Paramecia*’s swimming speed and the presence of five different organic and inorganic contaminants at varying concentrations centered around drinking water thresholds. The large size and ubiquity of the targeted microorganism, the avoidance of reagents or specialized tools for the measurement, and the simple data collection based on an object tracking algorithm enable the automatization of the assessment and real-time results using globally available technology.

## Introduction

Biomonitoring—that is, inferring the condition of an ecological system based on the observation of its organisms—is a central tool for ecosystem conservation, management, and restoration^1^. Most biomonitoring sampling methodologies were developed in the middle of the twentieth century, based on the then-existing ecological and technological knowledge. While considerable efforts are being made to modernize such measurements^2^, nowadays, most of those methods still consist of ecological censuses and taxonomic identifications, recording the diversity and abundance of multicellular organisms^3^. Despite the importance of this information to evaluate the impact and risks to humans and the environment, the data based on population changes do not necessarily capture the current state of the ecosystem but its past development.

Recording the population of microorganisms that are more sensitive to environmental changes and have shorter generation lengths than vertebrates, enables the use of methods such as the traditional half-maximal inhibitory concentration (IC_50_) to measure the effect of pollutants in water ecosystems in a few days^4^. However, as we progress in our understanding of environmental impact, the study of exogenous factors has moved beyond their influence on whole populations to include their effect on individual behaviors. Some of these abnormal behaviors resulting from the presence of pollutants have paved the way for the development of biomonitoring methods. The use of clams aperture is a recognized and extensively used technique to monitor in real-time the presence of heavy metals in water^5^. In the past few decades, other effects, such as fish assemblage^6^, movement and vocalization of birds^7^, or flying speeds in bees^8^, have been reported as possible air and water quality indicators.

While behavioral indicators enable the real-time acquisition of information^9^, their use is still hindered by their ambiguous nature, which might make their measurement using traditional biomonitoring methods extremely tedious, prone to error, or even impossible. However, as we develop new technologies, materials, and production models focused on their environmental impact^10^, obtaining more detailed and immediate information on their impact on the surrounding ecosystem is becoming even more critical^11^. On the other hand, the use of technology to obtain such field information on a global scale will necessitate the use of frugal engineering (i.e., avoiding specialized or expensive tools or consumables), such that the method is generalizable to resourceless, underdeveloped regions of the world^12^.

Here we demonstrate that the movement of *Paramecium aurelia* is a possible indicator for indirect measurement of chemical and physical properties of fluids, specifically for providing real-time information on the presence of pollutants in water. A simple object tracking algorithm enables us to obtain robust information in a few minutes. We demonstrate this correlation for several common organic and inorganic contaminants in the range of their permissible levels or limits (PL) in drinking water. Given the low technological requirements, the ubiquitous presence of *Paramecium* cells in fresh and sea water^13,14^, and the rapid output, requiring only a few minutes of video recording, the method developed is suitable for implementation in a broad range of situations and ecosystems.

## Results and discussion

### Experimental design

This study aims to identify the suitability of swimming patterns of the ubiquitous *Paramecia* to investigate the deviation in contaminated water due to the presence of pollutants. With a focus on the general applicability of the process, the approach is based on a frugal implementation that enables the measurement of the overall quality of water as a convolution of the impact of different additives on the swimming speed of aquatic microorganisms. Additionally, we also explore a scenario of constant chemistry and variable viscosity to use the changes in *Paramecium*’s swimming speed to indirectly quantify and document the environment’s rheology. Considering that the relation between the viscosity of an environment and the swimming speed of *Paramecia* has been manually measured multiple times in the past, here we use this magnitude to correlate the new automated methodology with previous results before its application to measure water contamination.

**Figure 1:**
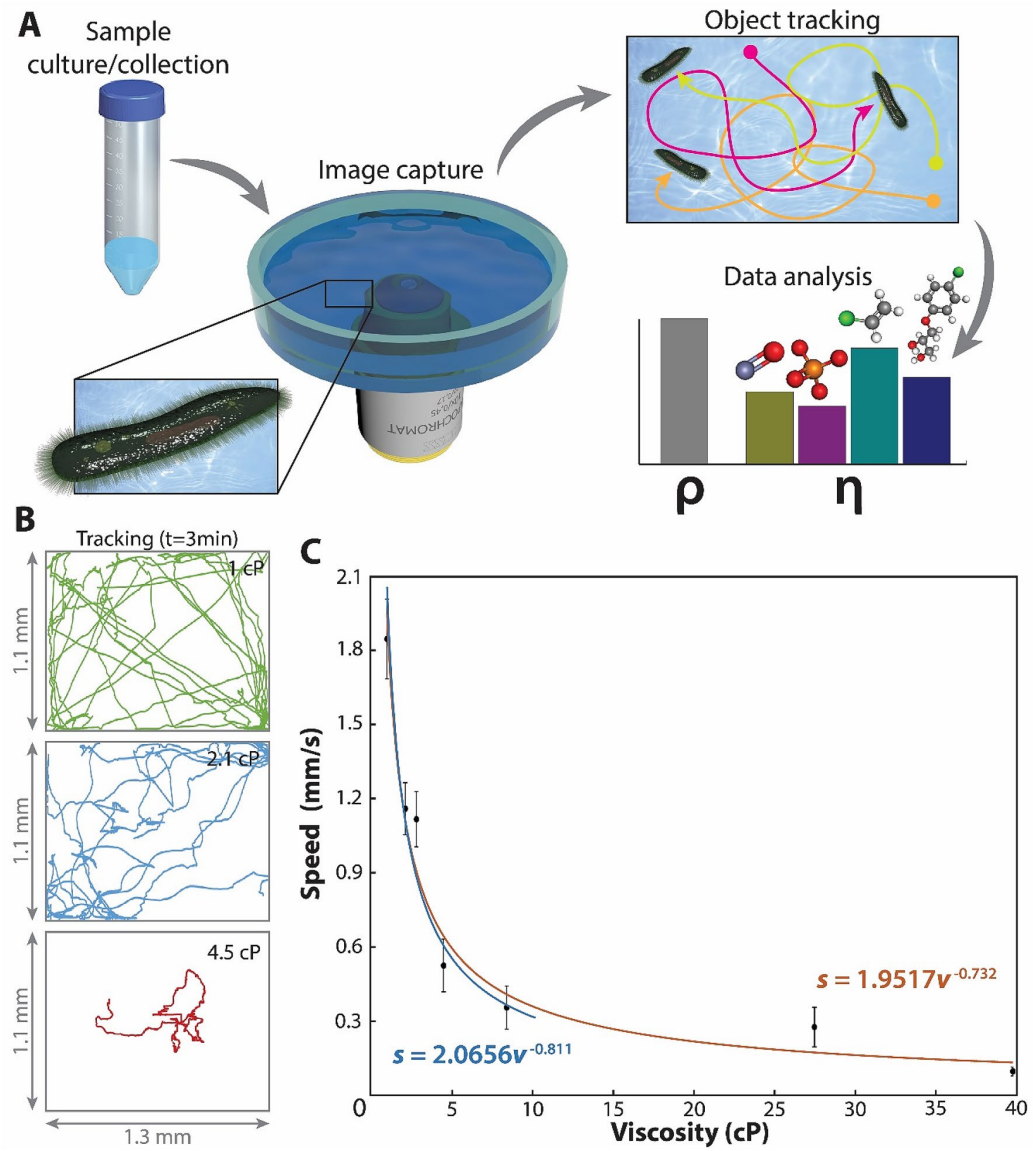
Automated tracking of *Paramecium* and measurement of viscosity. (**A**) The movement of *Paramecia Aurelia* cells in water samples is recorded for three minutes. Each cell is identified and followed through the video frames by an object tracking system. Their path is converted to cartesian coordinates, their speed calculated and correlated with changes in viscosity or presence of pollutants. Examples of path tracking are included in the Supplementary Fig. 1 and video 1. (**B**) Characteristics examples of the path followed by *Paramecia* for 3 min showing the whole field of view of the experiment conducted in water (top, green), 0.01% Methylcellulose (middle, blue), and 0.5% Methylcellulose (bottom, red). (**C**) Speed vs. viscosity for *Paramecia*. The red line is the fitting of the data to a power-law function for the whole range of viscosities tested (1–40 cP, R^2^ = 0.93). The blue line is the same fitting for the range of moderate viscosities (1–10 cP; R^2^ = 0.95). Error bars represent the standard deviation for the paths collected at each set of conditions (i.e., 30 *Paramecia* and paths per represented datapoint). Cumulative paths are presented in Supplementary Fig. 4.

The overall illustration of the study is shown in Fig. 1A. Samples containing free-swimming *Paramecia* were modified by the addition of Methylcellulose (MC) or with set concentrations of commonly occurring organic (i.e., erythromycin, tetrachloroethylene, trichloroethylene) and inorganic (i.e., zinc chloride and copper sulfate) pollutants. The movement of *Paramecia* was recorded for three minutes using a simple object tracking system based on a Kalman filter reported earlier^15^ (Supplementary video 1). The large size of *Paramecia*, reaching several hundreds of microns, enables the acquisition of information using low magnification lenses (i.e., 5x), by supplementing the consumer phone cameras with additional commercially available lens using ambient light^16^. The output of the *Paramecia* the tracking system (i.e., the position of each *Paramecium* cell in each video frame) recorded at 15 fps (i.e., 2,700 data points per *Paramecium* cell in the sample) was used to measure the distance traveled by and speed of each *Paramecium*.

It is noteworthy that similar studies to determine the migration of mammalian cells in environments of different mechanical characteristics concluded that path persistence is more significantly linked to the properties of the encapsulating environment than the migration speed^17^. In this case, the system was viewed on a 5 × objective lens to cover the geometrical constraints (i.e., a 1.1 × 1.3 mm chamber), necessary to fit *Paramecia* under a common charge-coupled device (CCD) field of view, forbid the calculation of those parameters as the collisions of *Paramecia* against the walls strongly affect those secondary observations. While those parameters might provide more granular information at the expense of more complex setups, as we demonstrate below, the use of swimming speed alone suffices for the quantitative measurement of viscosities and the qualitative presence of common pollutants, enabling practical application without any specialized devices.

### Indirect measurement of viscosity

In addition to the advantages of its ubiquity on water bodies of different nature, *Paramecium* cells possess three physical characteristics that make them a unique bioindicator from the perspective of technical implementation; as small (~ 0.05–0.32 mm) ciliated organisms, their swimming speed is strongly affected by the viscosity of the environment^18^ but not the temperature in the most common range of natural water (i.e., 5–20 °C)^19^, they are large enough to be easily observed with resource-poor microscopy setups^20^, and they swim independently of each other based only on hydrodynamic factors^21^.

The swimming speed of *Paramecia* in different viscosities has previously been measured manually several times^22,23^. While this study focuses on the first characterization of the influence of pollutants, the existence of previous literature on its relation with viscosity enables the comparison of our automated collection with other independent studies, and the evaluation of the robustness of *Paramecia*’s swimming speed for its use as a global indicator of water quality.

As observed in previous studies, our study corroborates that in the range of the low viscosity of 1–5 cP, *Paramecia*’s swimming speed is strongly influenced by the environment’s viscosity, showing in that range an average relative drop of 20.3% of the swimming speed at 1 cP per incremented cP (Figs. 1B and C, Supplementary Fig. 3, Supplementary video 2). Beyond 5 cP, *Paramecia*’s movement is hindered, losing significance at viscosities higher than ~ 10 cP. Fitting the data to the generally accepted power-law function^24,25^ provides an order of scaling of -0.732 (i.e., *s* = 1.9517*v*^-0.732^, where *s* is swimming speed and *v* is viscosity), which is significantly higher than that reported before. This discrepancy might be due to the consideration here of a more extensive range of viscosities. If only the 1–10 cP data are considered, the order of scaling decreases to -0.811, similar to the values obtained in previous experiments, which only consider that range of viscosities^22^. Therefore, the assumption of a power-law functional relationship between *Paramecium*’s swimming speed and a medium’s viscosity is more accurate in the low viscosity range (1–10 cP; R^2^ = 0.95), than when extreme cases are included (1–40 cP; R^2^ = 0.93). This can be easily explained due to the existence of a maximum viscosity when *Paramecia* movement is inhibited, a situation (i.e., swimming speed = 0) outside the boundaries of a finite power-law function.

The consistency of the data in the literature regarding the swimming speed of *Paramecia* and their dependence on the viscosity of the environment are noteworthy and suggest the suitability of the indicator for general use. Additionally, due to the self-motility and small size of *Paramecia*, this unconventional approach to obtain rheological information is particularly interesting for the measurement of microliter size, low viscosity biocompatible samples, a particularly challenging measure by conventional methods due to the inability to be inquired by regular rheometers or nanoindentations.

### Water pollutant detection

The correlation between viscosity and the swimming speed of *Paramecia* is a notable demonstration of the indirect measurement of the environment based on microorganisms’ behavior. The next step of the research evaluated the swimming speed variation of *Paramecia* with the chemical contamination of the environment. From the IC_50_ measurements, it is known that a microorganism’s reproductive capabilities are severely affected by pollution. Still, the few studies that focused on the effect of pollutants on swimming speed, performed in water tunnels with vertebrates, have demonstrated only a weak correlation between their exhaustion and water contamination^26^. In this study, the limitations of those previous works were circumvented by using an organism that is rapidly and largely affected by environmental changes and a parameter that can be immediately measured outside the laboratory conditions without human intervention (i.e., swimming speed). We analyzed *Paramecia*’s movement in various common water pollutants, such as families of heavy metals, chemical and pharmaceutical wastes, and volatile organic compounds (VOCs). We set the concentrations around the concentration limits in the country of this study (Singapore), which are similar to those prescribed by the World Health Organization^27^. To ensure that the response of the *Paramecia* is primarily biological and not physical/mechanical, we performed all the experiments at standard conditions (STP) and confirmed the limited impact in the viscosity of the pollutants, which reached a 9% increase when all five pollutants tested were simultaneously maxed at five times their PL (Supplementary table 4).

**Figure 2:**
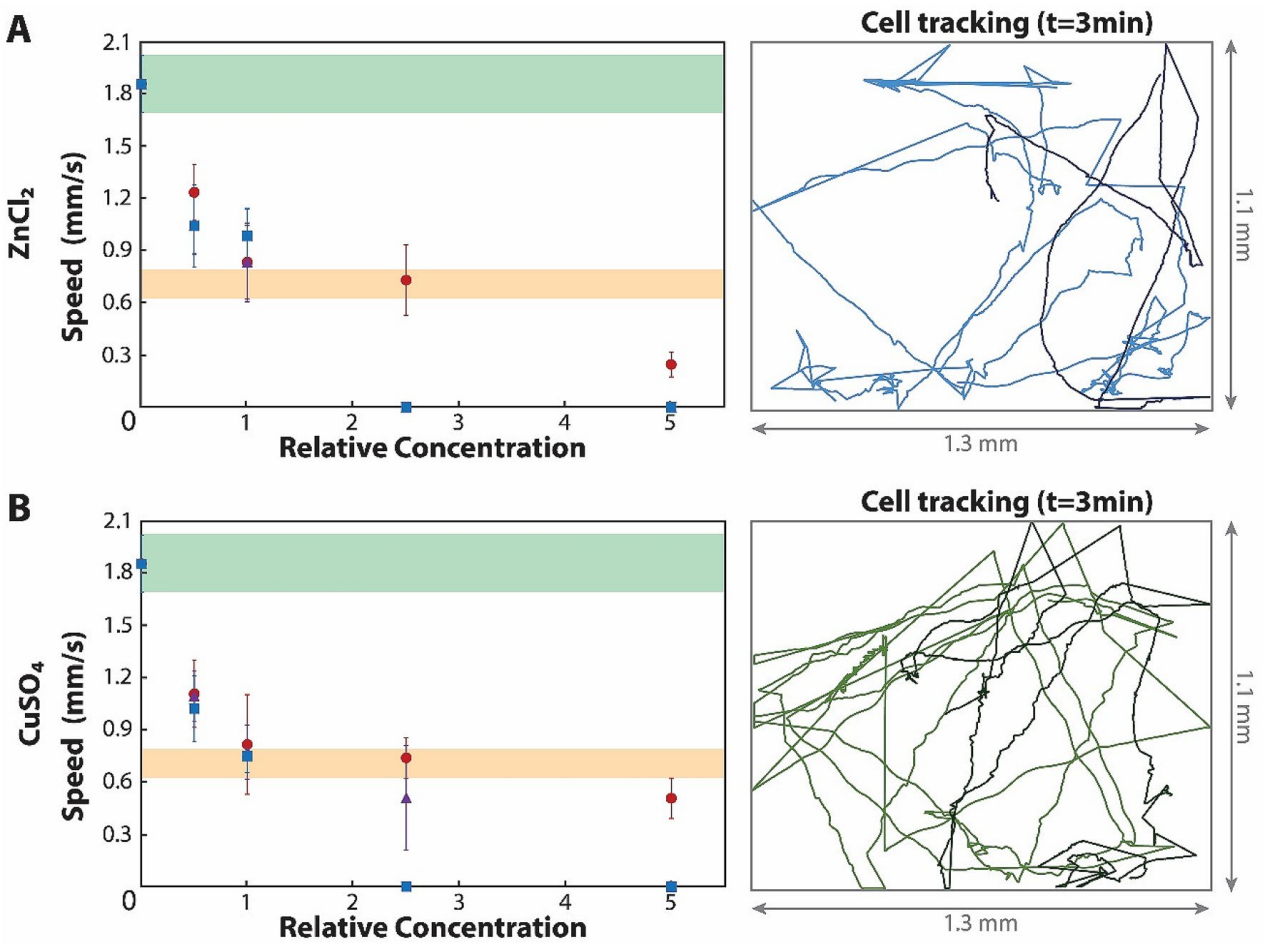
*Paramecia* in the presence of heavy metals. (**A**) Swimming speed of paramecia in different concentrations of zinc chloride (left). Red circular markers represent the speed immediately after the *Paramecia* are mixed in the Zinc chloride contaminated sample. Purple triangles and blue squares represent the average swimming speed of *Paramecia* after 24 and 48 h, respectively. Concentrations are relative to the PL (5 mg/l). The green and orange areas are the average and range of *Paramecia* speeds in unpolluted (green) and polluted (orange) media. The image on the right side is two characteristic paths followed by the *Paramecia* for 3 min at PL (light blue) and at five times PL (dark blue). Both are taken immediately after the *Paramecia* were mixed in the contaminated samples, and it represents the whole field of view. (**B**) Same representation than (**A**) but for copper sulfate. Markers use the same color code. PL, in that case, is 2 mg/l. The paths at the right side are for PL (light green) and five times PL (dark green). Error bars represent standard deviation of the average swimming speeds. Statistical significances are analyzed in Supplementary Fig. 2 and cumulative distances in Supplementary Fig. 4.

The presence of heavy metals immediately impacts *Paramecia*’s movement, showing a significant decrease in their speed even when zinc chloride (Figs. 2A) and copper sulfate (Fig. 2B) concentrations are only half of those considered unsafe for drinking water. This change happens in a time frame that enables real-time observations (Supplementary video 3). The average swimming speed immediately drops in both scenarios to almost half than in the unaltered media (to 66% for ZnCl_2_ and 59% for CuSO_4_). Further increasing the pollutant concentration does not proportionately increase the effect, but produces significant differences for samples with heavy metal concentrations at the safety limits. Interestingly, the hindered *Paramecia*’s swimming speed becomes stable with time, reaching the same constant slow swimming speed in the first 24 h for all the samples within permissible concentration levels. This may be due to the adjustment/adaptation of the microorganism to the surrounding presence of the chemical pollutants. Those concentrations beyond safe levels result in cell death in the first 24 h for all the samples, except for twice the threshold concentration of copper sulfate, when *Paramecia* death occurs between 24 to 48 h in the contaminated media.

Among all the pollutants tested, heavy metals showed the highest impact on the microorganisms’ swimming speed, resulting in death at concentrations immediately beyond those considered safe for drinking. This result could be interpreted as higher toxicity of heavy metals for *Paramecia* or a less strict regulation for heavy metal pollution with respect to that for organic pollutants tested in this study.

**Figure 3:**
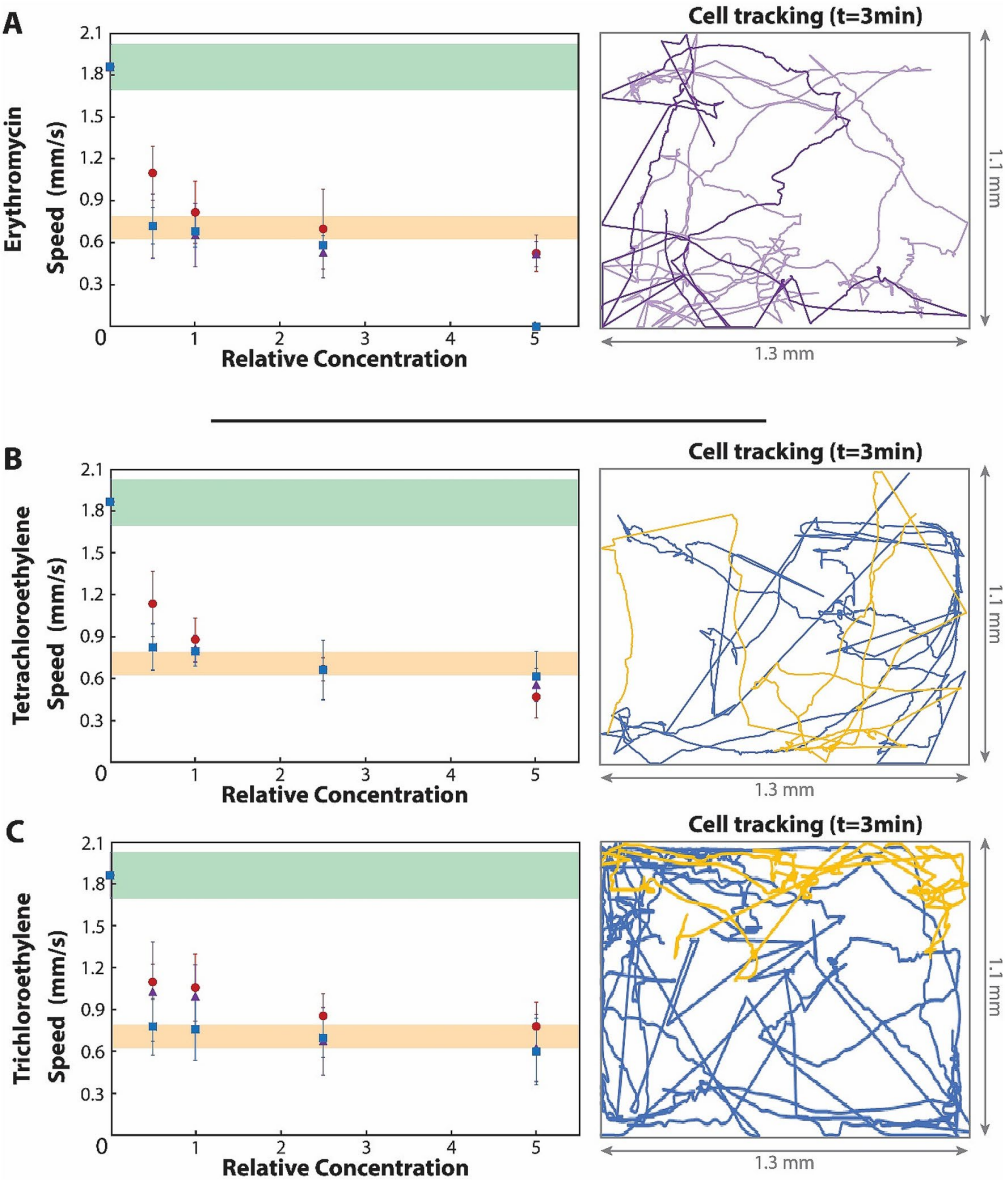
Measurement of the movement of *Paramecium* in the presence of organic pollutants. (**A**) Movement of *Paramecium* in Erythromycin solutions. The color code of the graph is similar to that in Fig. 2. The PL of erythromycin is 0.1 µg/l. Paths at the right side are for PL (light purple) five times PL (dark purple). (**B**) Swimming speed of *Paramecia* in samples contaminated with tetrachloroethylene (PL = 40 µg/l). Paths are for PL (blue) and five times PL (yellow). (**C**) Same representation but for trichloroethylene at (PL = 20 µg/l). Error bars represent standard deviation of the average speeds, and the paths are represented over the whole area of the chamber. Statistical significances are analyzed in Supplementary Fig. 2 and cumulative distances in Supplementary Fig. 4.

Similar patterns of swimming speed to those for heavy metals were observed from the impact of erythromycin, a common pollution indicator for an urbanizing global water cycle^28^ (Figs. 3A, Supplementary video 4). While erythromycin’s primary use is the selective treatment of bacterial infections, *Paramecia* also gets strongly affected by the antibiotic, which impacts its mitochondrial DNA^29^. However, in this case, cell death was only observed when erythromycin concentration reached five times the currently regulated limit. In the case of the VOCs tested (i.e., tetrachloroethylene and trichloroethylene, Supplementary video 5), none of them resulted in cell death even at the extreme concentrations tested, well beyond the safe limits (Figs. 3B and C).

**Figure 4:**
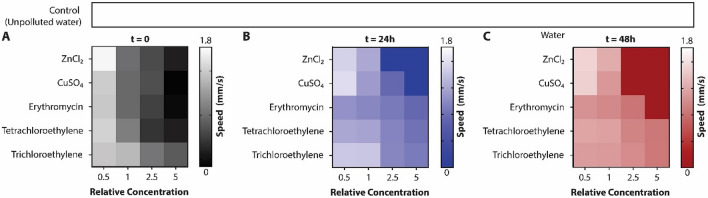
Swimming speed of *Paramecia* in all the pollutants tested. (**A**) Swimming speed immediately after their mixing in the contaminated samples. (**B**) Speed after 24 h in the contaminated sample. (**C**) Swimming speed 48 h in the contaminated samples. White is the average swimming speed of the *Paramecia* in the unpolluted reference environment.

There is a common pattern in all the pollutants tested (Fig. 4). There was an immediate and significant reduction in the swimming speed of *Paramecia* in all the pollutants at the concentrations considered an environmental/health hazard (Supplementary Fig. 2). *Paramecia* in all those samples where they were alive after 48 h achieved the same swimming speed (0.71 ± 0.08 mm/s), almost three times lower than the speed in uncontaminated water (1.86 ± 0.16 mm/s). Most of them reached this slow-swimming state in the first 24 h. The rate at which *Paramecia* reach this state of slow swimming speed depends on the concentration—reaching the state almost immediately in large concentrations, but taking up to two days in low concentrations. In the case of erythromycin and VOCs, this state is reached even at half the PL concentrations, while for heavy metals, the threshold is at the PL concentration.

This behavior raises two possible scenarios for using the swimming speed of *Paramecia* to assess water quality. The immediate and high relative change in *Paramecia*’s swimming speed suggests a suitable application to quickly evaluate, in a research environment, a specific pollutant’s impact. This type of test could, for example, substitute the standard IC_50_, based on reproducibility/mortality of the microorganisms, which is laborious, time-consuming and requiring trained personnel, reducing it to a few minutes of video recording, being the result also of a quantitative measurement with zero compromises on the quality.

Additionally, the existence of a stable and differentiable swimming speed in contaminated samples could be used as a rapid qualitative test field. In such situations, the suitability of a water sample could be assessed based on the average speed of the large microorganisms swimming in it and, in particular, those that are ubiquitous such as *Paramecia*.

## Conclusion

The swimming speed of *Paramecia* in a confined space is strongly correlated to environmental conditions, and it is easily measurable using a simple object tracking algorithm and CCD sensor. Inorganic and organic pollutants have been demonstrated to affect such speed significantly and consistently when they are present in concentrations around drinking water thresholds. The reliability of the parameters was studied using previous reports based on the viscosity of the environment, showing similar speeds and strong correlation in the range of 1–10 cP and also fitted to a power-law model within those boundaries.

The immediate impact of the presence of pollutants on the swimming speed and the persistence of that change for days suggest two modes of utilization of the magnitude: One, as a rapid alternative to traditional IC_50_ measurements in preestablished samples, dropping the required time from several days to few minutes; and the other, using the ubiquity of *Paramecia* in water bodies as an immediate and resource-poor field measurement to detect the presence of pollutants at levels that could pose a risk to human health.

## Material and methods

*Paramecium aurelia* was used for all the experiments in this study (Nantah Capital One Pte Ltd, Singapore). The *Paramecia* were further maintained in the laboratory as a mixed culture containing bacteria in a lettuce infusion, the latter prepared by brewing dried lettuce leaves in boiling water following a standardized protocol^30^. This media was used as a control/baseline to measure the swimming speeds of *Paramecia* in an unpolluted environment. Methylcellulose (MC) was purchased in a local store. The water pollutants used in the experiments (i.e., Zinc chloride, Copper sulfate, Erythromycin, Tetrachloroethylene, Trichloroethylene) were all purchased from Sigma-Aldrich (Germany). The polydimethylsiloxane (PDMS) is branded as SYLGARD 184 Silicone Elastomer Kit (Dow Chemical Company, USA) and was purchased from Tatlee Engineering Pte Ltd (Singapore).

### Sample preparation for rheology study

Different concentrations (w/v) of MC solutions (0.01%, 0.025%, 0.05%, 0.1%, 0.25% and 0.5%) in the reference media were prepared and filtered. The respective viscosity measurements were taken using the capillary viscometer and tabulated in Supplementary Table 1. Then 1 ml of a concentrated culture of *Paramecia* (by centrifuging at 300 × *g* ) was pipetted and incubated with 100 ml of the prepared samples with different viscosities in containers and stored on a bench-top at room temperature.

### Sample preparation for water pollutant detection study

The concentrations of the pollutants were chosen based on the concentrations of PL in water issued by PUB Singapore’s National Water Agency. The selected concentrations of 0.5x, 1x, 2.5x, and 5 × times the concentration of PL compared with 0x (water media control). The concentrations used were as follows: (1) Zinc chloride at 2 mg/l, 5 mg/l, 12.5 mg/l & 25 mg/l, (2) for Copper sulfate at 1 mg/l, 2 mg/l, 5 mg/l & 10 mg/l, (3) Erythromycin at 0.05 µg/l, 0.1 µg/l, 0.25 µg/l & 0.5 ug/l, (4) Tetrachloroethylene at 20 µg/l, 40 µg/l, 100 µg/l & 200 µg/l, and (5) Trichloroethylene at 10 µg/l, 20 µg/l, 50 µg/l & 100 µg/l. The stock solutions were prepared by diluting the pollutants in DI water, at the concentrations of 0.5 mg/ml (Zinc chloride), 1 mg/ml (Copper sulfate), 1 mg/ml (Erythromycin), 15 mg/ml (Tetrachloroethylene), and 17 mg/ml (Trichloroethylene). The testing solutions were prepared by adding stock solutions to the control media at the corresponding ratios. The “polluted” samples were then incubated with *Paramecia* (centrifuging at 300 × g and pipetted) in a process similar to that described above for viscosity measurements. The experiments were conducted at three time points: 0 h, 24 h, and 48 h at room temperature.

### Experimental setup

The experiments were carried out in an open well made up of PDMS with dimensions of 1.3 × 1.1 × 1 mm^3^ (w × l × h). From the samples prepared, *Paramecia* were pipetted out from the incubation container and transferred to the PDMS well, and their movements were recorded at a rate of 15 frames/sec using an inverted light microscope (Axio Observer D1, Carl Zeiss, Germany) and a custom-built camera at 5x, 0.15 NA during the experiment at room temperature. A customized MATLAB script (Math Work, USA) with its Vision and Image Analysis toolboxes was used to analyse the recorded videos^15^. All videos were processed frame by frame, and a Kalman filter with a path predictor was used to locate the microorganism and generate the data. A sample size of 30 *Paramecia* was used for each experimental condition, including the control samples, totaling approximately 2500 *Paramecia* for the rheology and water pollutant detection studies. Each *Paramecium* was considered a single object and assigned with a unique number as ID, and they were tracked frame by frame consecutively. Short-lived paths were automatically discarded as noise. The path prediction algorithm was used for more accurate tracking, enabling to track movement even in the events of losing the *Paramecium* for few frames (e.g., moving too close to the edge of the chamber, crossing with another *Paramecium,* or impurity in the media). All the tracked information about the generated path for each *Paramecium* was stored under the same ID in separate files as cartesian coordinates at each frame. With these obtained (x,y,t) values, total distance traveled and average swimming speed (= total path length/time) were calculated for each *Paramecium* (Supplementary Tables 2 and 3). Error bars in the figures represent the standard deviation for the averaged speed values of all the *Paramecia* used at each experimental condition (~ 30 cells).

## Supplementary Information

Supplementary Information 1.

Supplementary Video 1.

Supplementary Video 2.

Supplementary Video 3.

Supplementary Video 4.

Supplementary Video 5.
