# Supplementary material for: Measurements of the swimming speeds of motile microorganisms using object tracking and their correlation with water pollution and rheology levels
Source: Sci Rep. 2021 Jun 3;11:11821. doi: 10.1038/s41598-021-91134-1 (PMC8175393; doi:10.1038/s41598-021-91134-1)
Supplement: Supplementary file 1 — Supplementary Information 1. [file 41598_2021_91134_MOESM1_ESM.pdf]

Supporting Information

Measurements of the swimming speeds of motile microorganisms using object tracking and their correlation with water pollution and rheology levels

Ashaa Preyadharishini Shunmugam [a], Gowtham Subramanian [b] and Javier G. Fernandez [a]*

[a] Pillar of Engineering Product Development (EPD), Singapore University of Technology and Design (SUTD), Singapore 487372.

[b] Skin Research Institute of Singapore, Agency for Science Technology and Research, Singapore 138648.

* Corresponding author:
Dr. Javier G. Fernandez
E-mail: javier.fernandez@sutd.edu.sg

This document includes:

Figure S1 Examples of paramecia tracking.

Figure S2 Statistical significances for polluted environments (for Fig.2 and 3)

Figure S3 Swimming speed of individual paramecium in water.

Figure S4 Cumulative distances for the represented paramecia in figures 1 to 3

Table S1 Viscosity at different concentrations of Methyl Cellulose

Table S2 Travel distances in different polluted environments

Table S3 Travel distances and speeds respect t=0 in polluted environments

Table S4 Viscosity of the environment in extremely polluted environments

Video 1 Example of the *Paramecia* tracking

Video 2 *Paramecia* in biocompatible environments of different viscosities

Video 3 *Paramecia* in environments polluted with varying concentrations of heavy metals

Video 4 *Paramecia* in environments polluted with antibiotics (i.e., Erythromycin)

Video 5 *Paramecia* in environments polluted with volatile organic compounds.

SFig.1

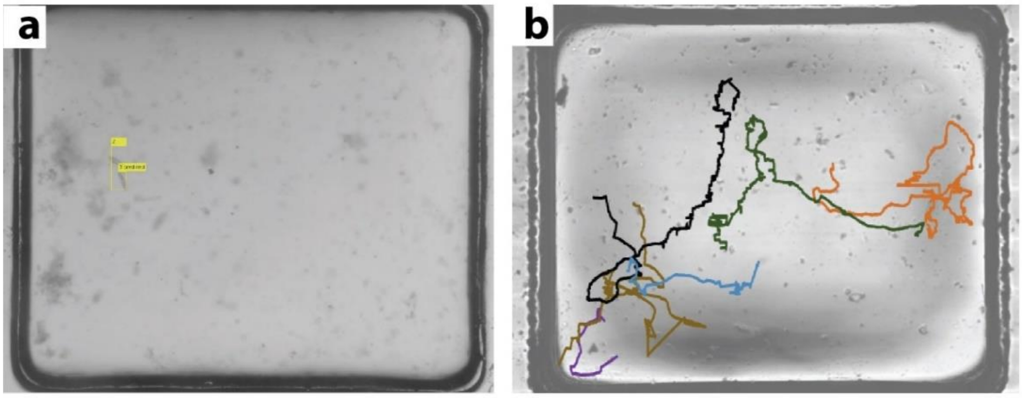

Supplementary Figure 1. *Paramecium* tracking. (**A**) Image showing the Object (*Paramecium*) recognition by MATLAB for data acquisition. Each *Paramecium* will be assigned a unique number ID displayed in the yellow box (**B**) Image displaying a depiction of the paths travelled by six *Paramecia* inside the well. Each colour corresponds to a single *Paramecium* path.

SFig.2

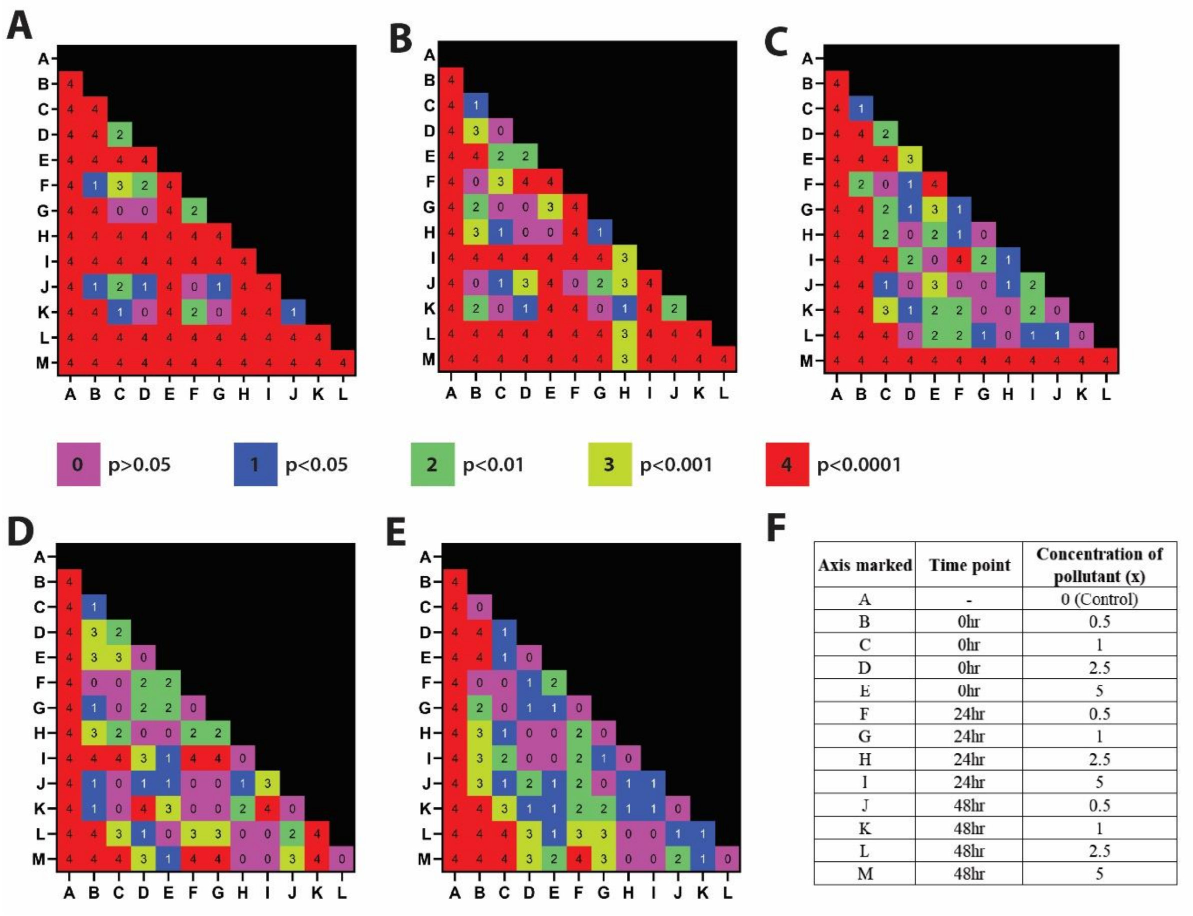

Axis marked	Time point	Concentration of pollutant (x)
A	-	0 (Control)
B	0hr	0.5
C	0hr	1
D	0hr	2.5
E	0hr	5
F	24hr	0.5
G	24hr	1
H	24hr	2.5
I	24hr	5
J	48hr	0.5
K	48hr	1
L	48hr	2.5
M	48hr	5

Supplementary Figure 2. Statistical significance for polluted environments. Statistical analyses between the swimming speed of *Paramecia* in the polluted media at different concentrations and timepoints through ANOVA Analysis (one-way ANOVA) with alpha value at 0.05. **(A)** Zinc chloride, **(B)** Copper sulfate, **(C)** Erythromycin, **(D)** Tetrachloroethylene, and **(E)** trichloroethylene. **(F)** Letter codes for the axes in (A-E). The second column is timepoints (t=0, 24h, and 48h) and the third concentration; the latter expressed as a fraction of the Permissive Levels (PL). Color/numbers represents the p-value with 0/purple being $p>0.05$ (i.e., not significant), 1/blue $p<0.05$, 2/dark green $p < 0.01$, 3/light green $p < 0.001$, and 4/red $p< 0.0001$.

SFig.3

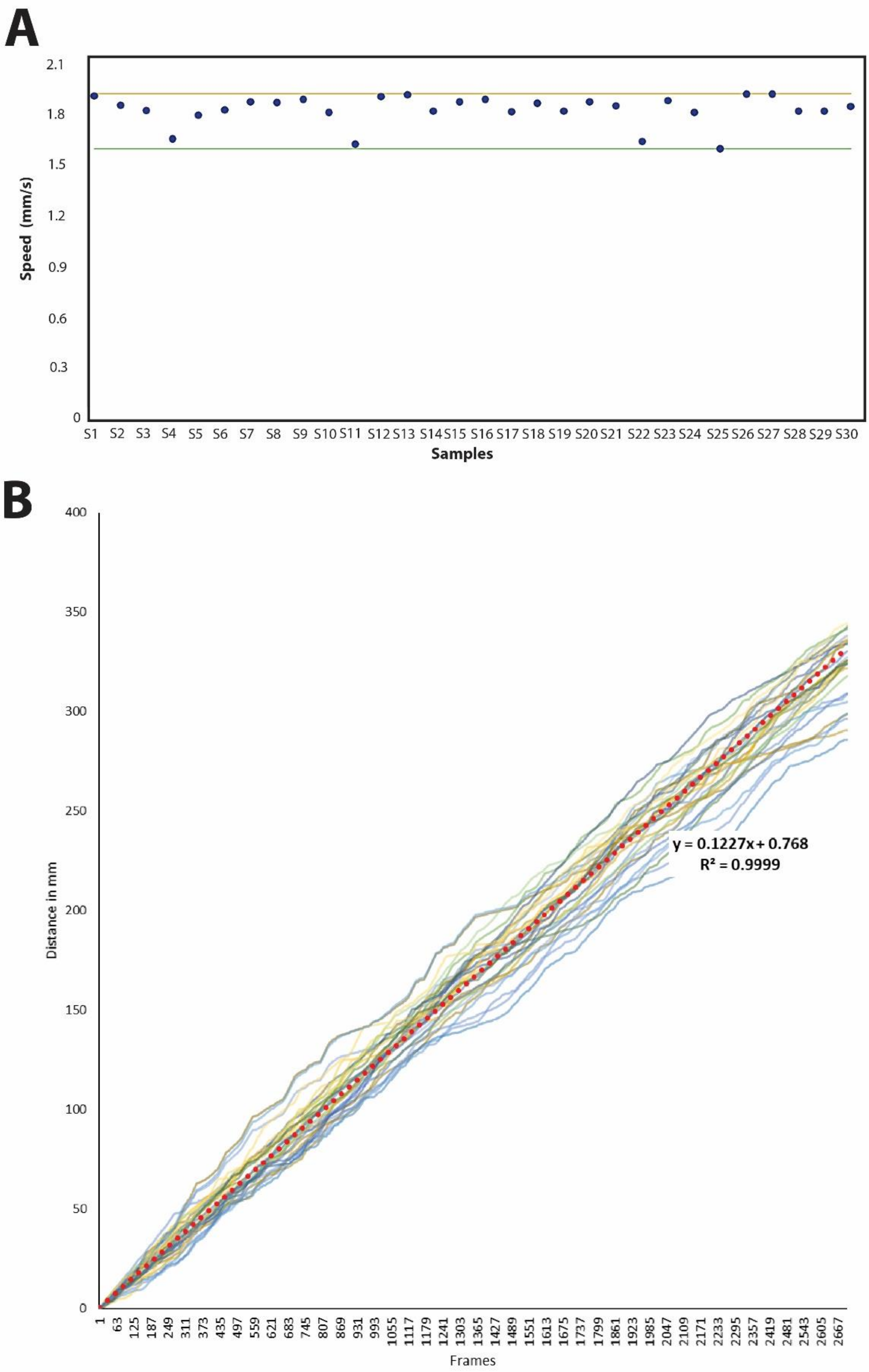

Supplementary Figure 3. (A) Speed of 30 Paramecia in water (Control). **(B)** Cumulative distances in the three minutes of testing.

SFig.4

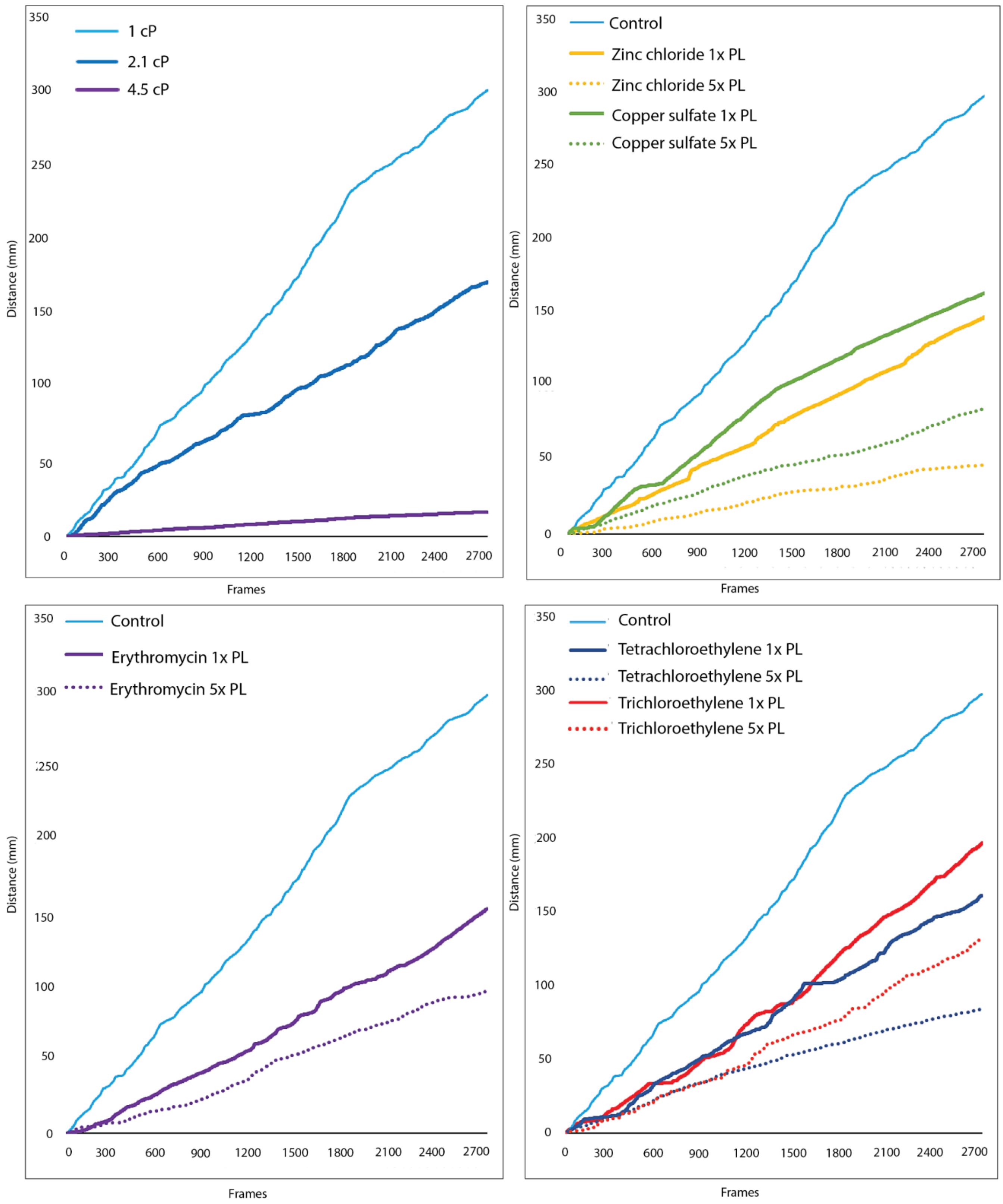

Supplementary Figure 4. Cumulative distances (in mm) for all the *Paramecia*'s paths in Figures 1 to 3. The horizontal axis represents frames, recorded at 15Hz.

Supplementary Table S1

Table 1. Viscosity of methylcellulose (MC) solutions Different concentration of the methylcellulose solutions and their respective viscosities

MC concentration	Relative Viscosity of MC samples
0.01%	2.135135
0.025%	2.835381
0.05%	4.528256
0.1%	8.461916
0.25%	27.63882
0.5%	40.0

Supplementary Table S2

Table 2a. Shows the percentage decrease of the distance traveled by the *Paramecium* in the different water pollutant samples

% decrease	0-hr				24-hr				48-hr			
	0.5x	1x	2.5x	5x	0.5x	1x	2.5x	5x	0.5x	1x	2.5x	5x
**Zinc**	32%	54%	60%	67%	43%	54%	-	-	43%	53%	-	-
**Copper**	39%	55%	59%	72%	40%	57%	72%	-	44%	59%	-	-
**Erythromycin**	40%	55%	62%	71%	60%	63%	67%	72%	60%	63%	68%	-
**Tetrachloroethylene**	38%	52%	64%	67%	54%	55%	64%	68%	55%	56%	64%	68%
**Trichloroethylene**	40%	42%	53%	57%	45%	46%	63%	66%	57%	58%	62%	67%

Table 2b. Shows the average percentage decrease of the distance of the *Paramecium* movement at different concentration of water pollutant samples

Average	0.5x	1x	2.5x	5x
**Zinc**	39%	54%	60%	67%
**Copper**	41%	57%	66%	72%
Average of heavy metals	40%	55%	63%	69%
**Erythromycin**	54%	60%	65%	71%
**Tetrachloroethylene**	49%	55%	64%	68%
**Trichloroethylene**	48%	49%	59%	63%
Average of VOCs	48%	52%	62%	65%

Supplementary Table S3

Table 3a. Shows the percentage decrease of the distance of the *Paramecium* movement at different time points of done with respect to the 0-hr time point

Samples		Distance % difference			Speed % difference		
		**0 hr**	**24 hr**	**48 hr**	**0 hr**	**24 hr**	**48 hr**
**Zinc**	**0.5x**	0%	18%	18%	0%	18%	18%
	**1x**	0%	0%	3%	0%	0%	15%
	**2.5x**	0%	100%	100%	0%	100%	100%
	**5x**	0%	100%	100%	0%	100%	100%
**Copper**	**0.5x**	0%	1%	8%	0%	1%	8%
	**1x**	0%	6%	9%	0%	6%	9%
	**2.5x**	0%	45%	100%	0%	45%	100%
	**5x**	0%	100%	100%	0%	100%	100%
**Erythromycin**	**0.5x**	0%	53%	52%	0%	53%	52%
	**1x**	0%	21%	20%	0%	25%	20%
	**2.5x**	0%	15%	20%	0%	32%	20%
	**5x**	0%	1%	100%	0%	1%	100%
**Tetrachloro- ethylene**	**0.5x**	0%	36%	38%	0%	37%	38%
	**1x**	0%	8%	11%	0%	8%	11%
	**2.5x**	0%	1%	1%	0%	1%	1%
	**5x**	0%	5%	3%	0%	16%	24%
**Trichloro- ethylene**	**0.5x**	0%	10%	41%	0%	7%	41%
	**1x**	0%	7%	39%	0%	6%	39%
	**2.5x**	0%	27%	23%	0%	27%	23%
	**5x**	0%	25%	30%	0%	25%	30%

Table 3b. Shows the percentage decrease of the distance of the *Paramecium* movement comparing the 24-hr and 48-hr time point

Samples		Distance % difference	Samples		Distance % difference
**Zinc**	**0.5x**	0%	**Tetrachloroethylene**	**0.5x**	1%
	**1x**	3%		**1x**	2%
	**2.5x**	0%		**2.5x**	0%
	**5x**	0%		**5x**	2%
**Copper**	**0.5x**	7%	**Trichloroethylene**	**0.5x**	22%
	**1x**	3%		**1x**	30%
	**2.5x**	0%		**2.5x**	3%
	**5x**	0%		**5x**	4%
**Erythromycin**	**0.5x**	0%			
	**1x**	1%			
	**2.5x**	5%			
	**5x**	100%			

Supplementary Table S4

Table 4 Viscosity measurements of the pollutants

Time	Relative viscosity	Increase %
**Control (water)**	1	NA
**All (5) pollutants (all at 5×PL)**	1.097902	9.79%
**Zinc (5×PL)**	1.083916	8.39%
